# Creep Characteristics of Layered Rock Masses after Water Absorption Due to Structural Effects

**DOI:** 10.3390/ijerph20054055

**Published:** 2023-02-24

**Authors:** Huichen Xu, Xiaoming Sun, Yong Zhang, Chengwei Zhao, Chengyu Miao, Dong Wang

**Affiliations:** 1 College of Mechanical and Architectural Engineering, Taishan University, Taian 271000, China; 2 State Key Laboratory for Geomechanics and Deep Underground Engineering, China University of Mining and Technology, Beijing 100083, China; 3 School of Mechanics and Civil Engineering, China University of Mining & Technology (Beijing), Beijing 100083, China; 4 State Key Laboratory of Mining Disaster Prevention and Control Co-Founded by Shandong Province and the Ministry of Science and Technology, Shandong University of Science and Technology, Qingdao 266590, China

**Keywords:** water content, structural effect, acoustic emission energy, layered rock mass, creep characteristic

## Abstract

Affected by the “three highs and one disturbance” (high ground pressure, high ground temperature, high permeability pressure, and strong mining disturbance), deep layered rock mass roadways often display large deformations, resulting in accidents and disasters from time to time. This paper aims to study creep characteristics of layered rock masses after water absorption due to structural effects, combined with acoustic emission energy and dominant frequency value analysis. Experimental results show that as the water content decreases, the long-term strength of the rock sample increases, and the damage becomes more severe. Under the same water content state conditions, the rock samples with bedding angles of 0°, 30°, and 90° have high long-term strength and undergo severe failure, whereas rock samples with bedding angles of 45° and 60° have low long-term strength and undergo mild failure. Under the same water content, the initial energy release increases with the bedding angle. Under the same water content, the energy release during failure decreases first and then increases with the increasing bedding angle. The initial energy, the cumulative energy, the initial main frequency, and the main frequency at the time of failure tend to decrease with the increase in water content.

## 1. Introduction

The deep rock mass produces a large number of structural planes during its long formation process under the action of geological factors. Due to the special nature of structural planes, their development state, expansion level, mechanical properties, and distribution laws directly affect the strength and the physical and mechanical properties of the rock mass [1,2,3,4,5]. A layered rock mass is a special rock mass with one or more sets of structural planes, and its lithology often exhibits the typical mechanical characteristics of large plastic deformation, strong anisotropy, and low long-term strength. Slate is a typical representative of a layered rock mass. Affected by the complex geomechanical environment of deep “three highs and one disturbance” (high ground pressure, high ground temperature, high permeability pressure, and strong mining disturbance) [6,7], deep-layered rock mass roadways often display large deformations and shrinkage of two sides and roofs, resulting in accidents and disasters from time to time. The characteristics of large deformation and failure of nonlinear creep have obvious rheological characteristics [8,9,10,11,12,13,14,15]. The reason is that, during the excavation process of the deep layered rock mass roadway, it is caused by its different bedding angles to produce structural effects, coupled with the adsorption of water in the high-humidity environment, resulting in long-term strength reduction.

At present, many scholars have conducted much work in the study of the mechanical properties of layered rock masses under the influence of rock mass structural planes and influencing factors [16,17,18,19,20,21,22,23,24,25,26], such as Hou et al. [27], who used shale with different bedding angles to conduct Brazilian splitting experiments. With the help of a high-speed photography system and an acoustic emission system, the mechanical properties of shale, crack propagation law, and acoustic emission characteristics were shown to change with the bedding dip angle.

However, in view of the characteristics of nonlinear creep and large deformation failure caused by water absorption and creep of deep layered rock masses, considering the two influencing factors of water content and structural effect, combined with acoustic emission energy and dominant frequency value (DFV) analysis, indoor experiments, microstructure analysis, and test methods used to study the creep of layered rock masses are rarely reported. According to the laws of change of the variable mechanical properties, a creep constitutive model that considers the water content and structural effects is established to reveal the water absorption and softening mechanism of layered rock masses. The study on deep layered rock mass rheology provides a theoretical and experimental basis for roadway engineering disaster control with sufficient engineering and geological background, and has important theoretical and practical significance.

## 2. Engineering Background

### 2.1. Engineering Geological Background

**Figure 1:**
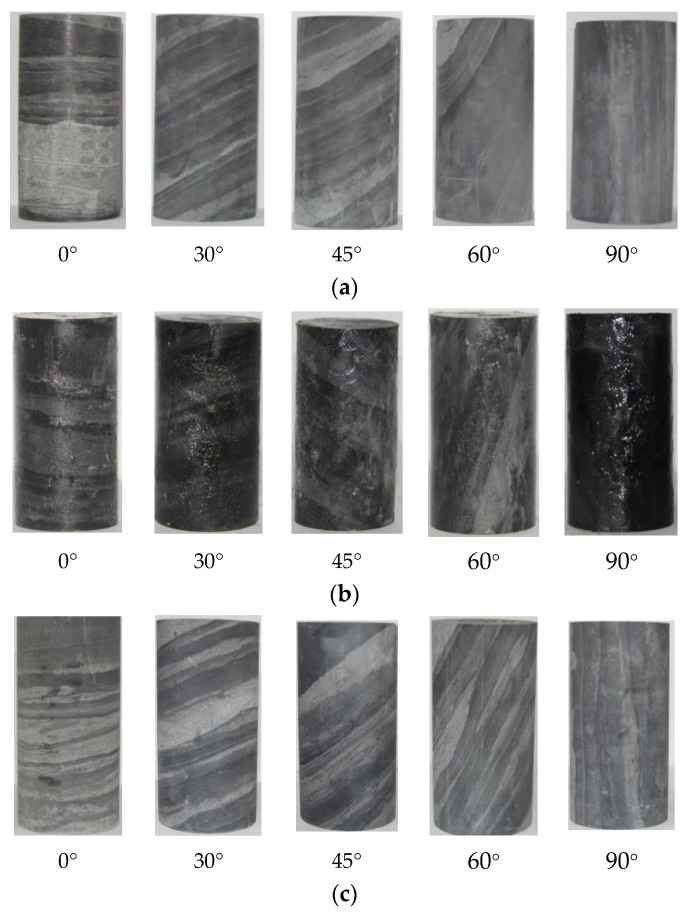
Rock samples after processed: (**a**) under natural state; (**b**) saturated state; (**c**) dry state.

The slate samples were taken from the start and end pile numbers of the Muzhailing deep-buried tunnel, which are AZK216 + 380~AZK220 + 300. The tunnel is a large-buried and extra-long highway tunnel with a maximum buried depth of approximately 638 m and a maximum horizontal principal stress of 24.95 MPa. The surrounding rock of this tunnel section is a shallow metamorphic slate, which is a kind of layered rock mass. The surrounding rock fissures are relatively developed. Many rock masses are broken, conjoined with the action of the groundwater. Due to the long-term action of groundwater, the surrounding rock at the site has the failure characteristics of arch deformation and large local vault deformation. On-site rock samples with different angles were taken for processing (e.g., 0°, 30°, 45°, 60°, and 90°). The distance of on-site rock samples with 0°, 30°, 45°, 60°, and 90° taken from the start pile was almost 310 m, 560 m, 610 m, 820 m, and 1310 m, respectively. The processed rock samples are shown in Figure 1.

### 2.2. Microstructure Analysis

**Figure 2:**
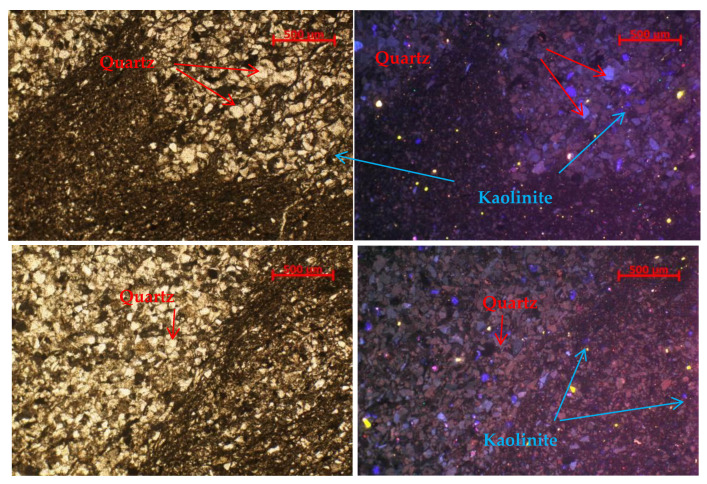
Cathodoluminescence picture near the beddings for slate.

A rock sample was taken for cathodoluminescence scanning. From the picture, the slate near the bedding plane is divided into muddy and sandy parts. In the muddy part, the fine quartz fragments are mainly blue–purple, with a small amount of brown–red. The few fine feldspar fragments are mainly blue. Kaolinite is indigo blue and dispersedly distributed. In the sandy part, the detrital quartz is mainly blue–purple, followed by brown–red. A small amount of feldspar can be seen, blue in color. Kaolinite is indigo blue. Below the cathode, the quartz fine debris is mainly blue–violet, with a small amount of brown–red, and a small amount of feldspar fine debris, mainly blue, and kaolinite indigo blue, scattered (Figure 2).

**Figure 3:**
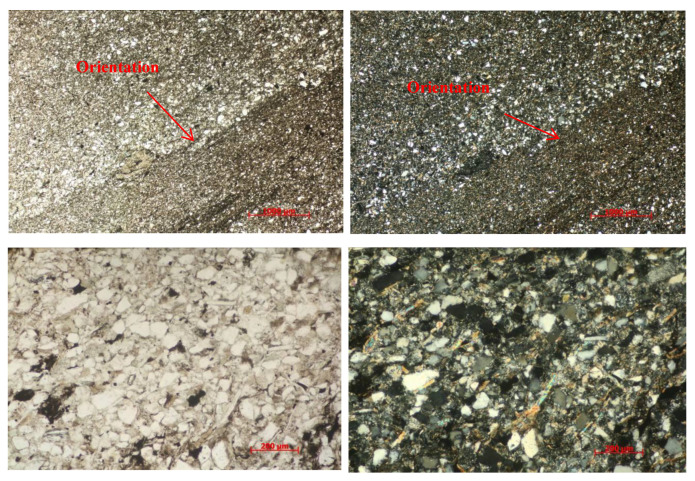
Thin sheet of casting for slate.

A thin sheet of casting for slate near the beddings is shown in Figure 3. The silt-ultrafine-grained sand-like structure has an obvious bedding structure. The sand and mud are slightly interbedded, and the clastic line-point contact is dominant. The clastics are mainly composed of quartz, feldspar, and mica, with a small amount of rock fragments. The particle size of the clastics is mainly between 0.01 and 0.12 mm, with a maximum of 0.22 mm, thereby forming a porous cementation. The mica is arranged in a directional arrangement; part of the mud is interstitial and partly distributed in layers, and calcite is dispersed.

### 2.3. Mineral Content Analysis

**Figure 4:**
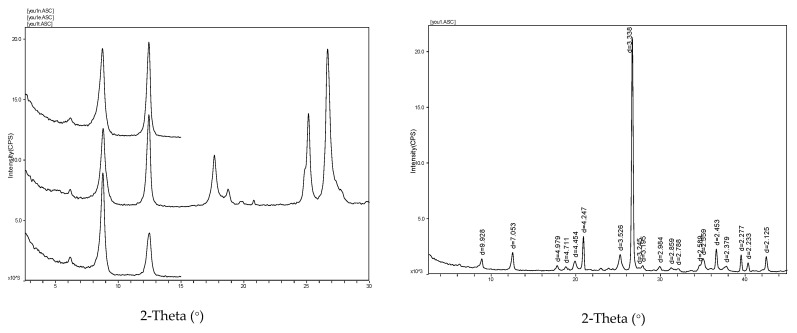
Mineral analysis spectrogram.

The rock samples were taken for X-ray derivation. The results are shown in Figure 4. The rock samples are mainly composed of quartz and clay minerals. Quartz accounts for 49.6%, and clay minerals account for 47.9%. The amount of clay minerals is relatively high. The main types of clay minerals are illite, chlorite, illite–Montmorillonite, and kaolinite. The analysis of mineral composition shows that the tunnel rock sample has a high clay mineral content, within which the content of the illite–Montmorillonite mixed layer is relatively high. The illite–montmorillonite mixed layer has strong water absorption, and the volume expands sharply after absorbing water, which easily causes softening, disintegration, and expansion, thereby reducing the strength of the rock.

## 3. Experimental Method

Slate samples were processed into Φ50 mm × 100 mm standard rock samples, and the sample height and diameter error were controlled within ±0.2 mm. We selected 30 samples for basic experiments, and 15 samples for creep experiments. The whole procedure was carried out according to the standard. The saturated water-absorbent rock sample was obtained by the immersion method. The specific method aims to completely immerse the rock sample in distilled water and weigh it every day. When the increase in the rock sample approached 0, the water absorption experiment was stopped, and the average water content of the saturated rock sample was measured as 1.3%. The dry rock sample was obtained by drying for 24 h through a drying device, and the average moisture content of the natural rock sample was measured as 0.6%.

In view of the long loading time, to avoid the evaporation of water during the loading process, the rock sample was sealed after saturated water absorption; the surface was coated with petroleum jelly. The natural, saturated, and dry rock samples are shown in Figure 1. The creep experiment was divided into three groups of experiments according to different water-bearing states and rock inclination angles (e.g., 0°, 30°, 45°, 60°, and 90°). The test first performed uniaxial compression tests on rock samples with different water-bearing states. Then, a creep experiment was conducted. The creep experimental program is shown in Table 1.

**Figure 5:**
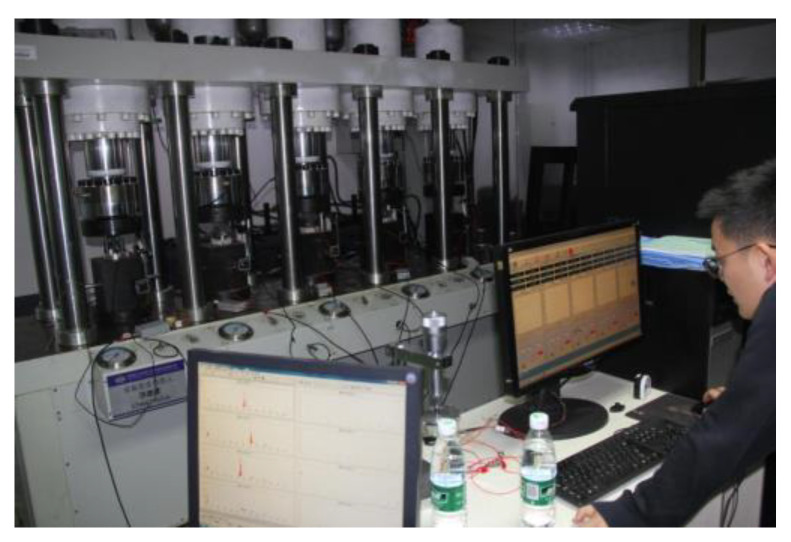
The five-link creep experiment system.

The experiment adopts the five-link creep experiment system, which mainly includes the (1) host, (2) environmental device, (3) power source, and (4) microcomputer measurement and control device (Figure 5).

The creep experiment adopts a graded loading method. To facilitate the analysis of stress creep characteristics, the experiment adopts the same stress loading path. The uniaxial compressive strength of the rock sample with a bedding dip angle of 45° was selected as the instantaneous strength standard, and the loading stress level is shown in Table 2.

**Figure 6:**
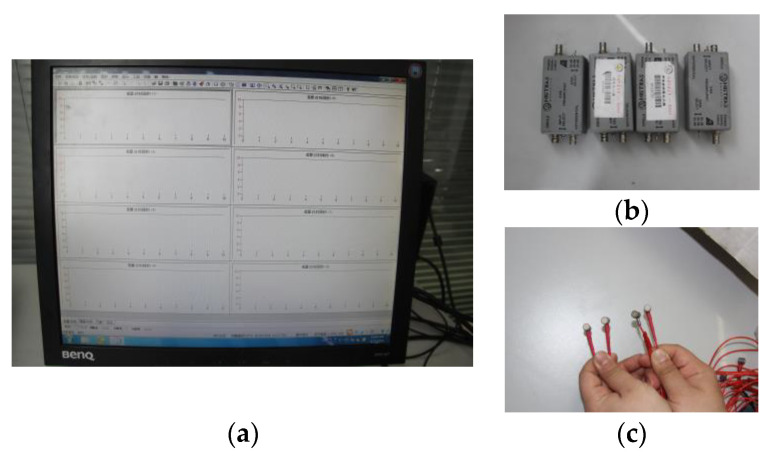
Acoustic emission system. (**a**) acoustic emission acquisition system; (**b**) amplifier; (**c**) sensor.

An acoustic emission monitoring system was used as an auxiliary experiment. The acoustic emission in the experiment was carried out using the PCI-2 type. Its basic parameters are a frequency response range of 100 Hz–1 MHz, a sampling rate of 2 M effective data points per second, and a threshold of 50 dB. The acoustic emission acquisition system, amplifier, and sensor are shown in Figure 6.

**Figure 7:**
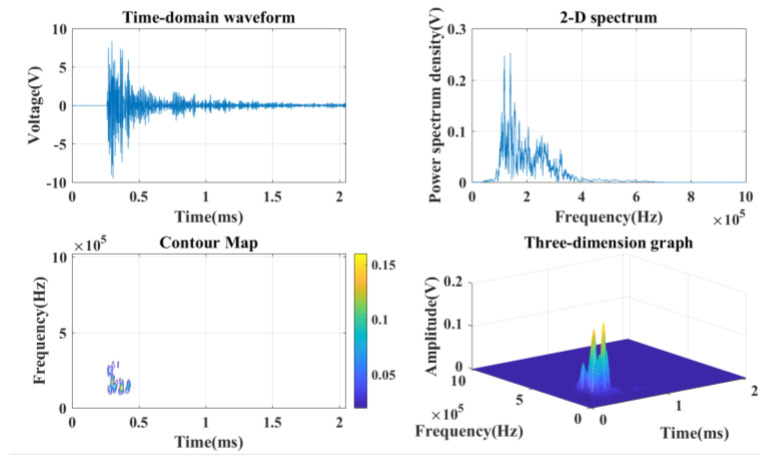
Time–frequency feature map.

The fast Fourier transform (FFT) is used to convert continuous random signals from the time domain to the frequency domain to complete the analysis of waveform signals by converting them into the frequency domain during the loading process of rock samples. The acoustic emission signal is formed into a waveform signal file every 4096 numbers, and the acquisition time is 2 ms. After the waveform file is formed, the three-dimensional amplitude-frequency-time spectrum is obtained by using the fast Fourier transform and the short-term Fourier transform by MATLAB, as shown in Figure 7. The quick Fourier transform is calculated as follows [28]:

(1) $$X(k)=\sum_{i=0}^{N}x(i)e^{-j2\pi ik/(N+1)}\;(k=0,\;1,\;2,\;......,\;N)$$

The squared expression for FFT is:

(2) $$S_{n}(t,f)={\left|\int_{-\infty }^{+\infty }N(t)\eta (t_{1}-t)e^{-i2\pi ft_{1}}dt_{1}\right|}^{2}$$

## 4. Experimental Results

### 4.1. Results of Uniaxial Compressive Strength

**Figure 8:**
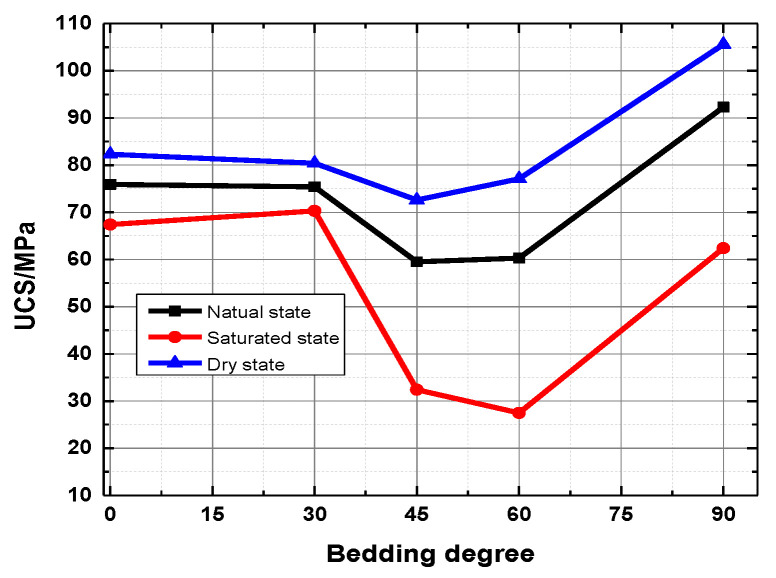
The relationship of bedding angles and uniaxial compressive strength.

**Figure 9:**
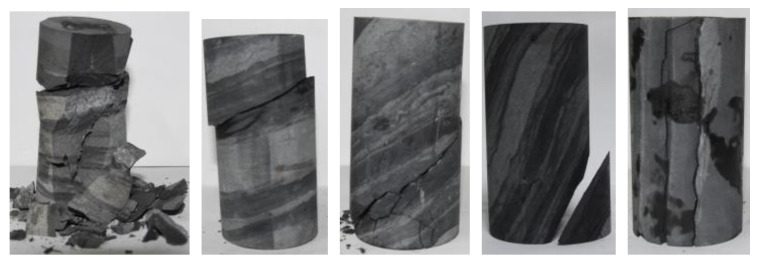
Typical failure picture of rock sample under saturated state.

The uniaxial compressive strength of the rock sample is not significantly reduced in the natural state compared with the dry state. The reason is that the clay mineral reaction is not evident when the water content is low, especially when the weak surface between the layers is less affected. The uniaxial compressive strength did not decrease significantly. The insignificant reaction of clay minerals in low water content rock samples has minimal effect on the weak bedding surface. Compared with the rock samples in the saturated state, the uniaxial compressive strength of the rock samples with different bedding dips in the dry state and the natural state do not differ greatly. In the saturated state, the clay minerals and the interlayer weak surface filling fully absorb water and expand, thereby resulting in a sharp drop in the uniaxial compressive strength of the slate, especially for the rock samples with a bedding dip of 45° and a bedding dip of 60°. Figure 8 shows the relationship between bedding angles and uniaxial compressive strength. Figure 9 is a typical failure picture of a rock sample under a saturated state.

### 4.2. Creep Experiment Result Analysis

**Figure 10:**
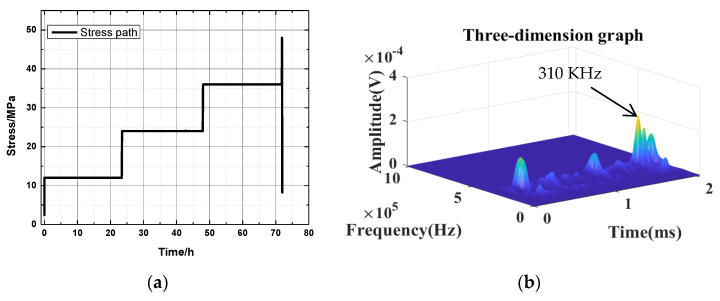

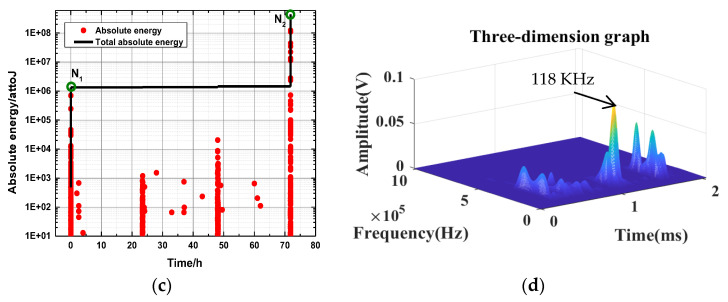
Stress with time for the 45 bedding angle under natural state: (**a**) the actual stress path; and (**c**) the corresponding acoustic emission energy curve. Images (**b**,**d**) are the waveform characteristics of key points.

Only a portion of the experimental results are displayed, because of space limitations. Stress with time for 45 bedding angles under the natural state is taken as a typical example (Figure 10). Figure 10a shows the actual stress path of the creep experiment, Figure 10c presents the corresponding acoustic emission energy curve, and Figure 10b,d illustrate the waveform characteristics of key points, that is, the dominant frequency characteristic values corresponding to N1 and N2 in Figure 10c. Point N1 corresponds to the DFV at the end of the first loading, and point N2 corresponds to the DFV at the time of failure.

Table 3 displays the AE energy level for different bedding angles under the natural state. AE energy is usually measured using mean square voltage, defined as follows:

(3) $$V_{ms}=\frac{1}{\Delta T}\int_{0}^{\Delta T}V^{2}(t)dt$$

where Δ*T* is the average time, *V*(*t*) is the signal voltage of time change, *V_ms_* is the change rate of of acoustic emission energy, and the total energy *E* of the acoustic emission from time *t*_1_ to *t*_2_ can be expressed by the following equation:

(4) $$E=\frac{1}{\Delta T}\int_{t_{1}}^{t_{2}}V_{ms}dt$$

Table 4 shows the DFV of the key point for different bedding angles under the natural state. Table 3 shows that the accumulated energy levels of key point N_1_ with different bedding angles under natural conditions are between 3.9 × 10^4^–6.6 × 10^5^. The cumulative energy level of the key point N_2_ is between 2.4 × 10^8^ and 1.8 × 10^9^. Table 4 shows that the key points N_1_ to N_2_ of rock samples with different bedding angles under natural conditions change from low amplitude to high amplitude, and the amplitude increases significantly.

**Figure 11:**
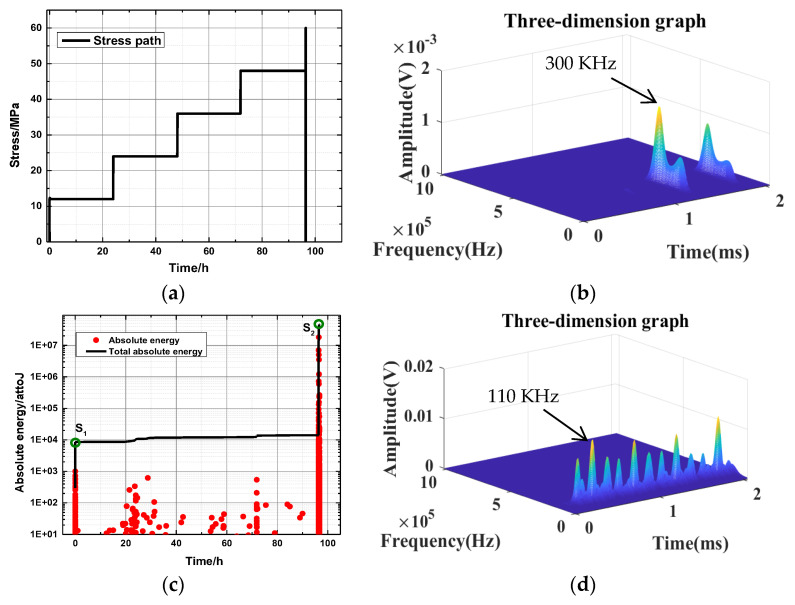
Stress with time for a 30-degree bedding angle under the saturated state: (**a**) the actual stress path; and (**c**) the corresponding acoustic emission energy curve. Images (**b**,**d**) are the waveform characteristics of key points.

Stress with time for a 30-degree bedding angle under the saturated state is taken as a typical example, as shown in Figure 11. The AE energy levels for different bedding angles under the saturated state are shown in Table 5. Table 6 shows the DFV of the key point for different bedding angles under the saturated state. Failure occurs when bedding angles of 45° and 60° are loaded to the first stage (12 MPa), which is not analyzed in this part. Table 5 shows that the accumulated energy levels of key point S_1_ with different bedding angles under saturation conditions are between 2.7 × 10^3^ and 3.6 × 10^4^. The cumulative energy level of key point S_2_ is between 4.1 × 10^7^ and 1.1 × 10^8^. Table 6 shows that the key points S_1_ to S_2_ of rock samples with different bedding angles change from low amplitude to high amplitude under natural conditions, and the amplitude increases significantly.

**Figure 12:**
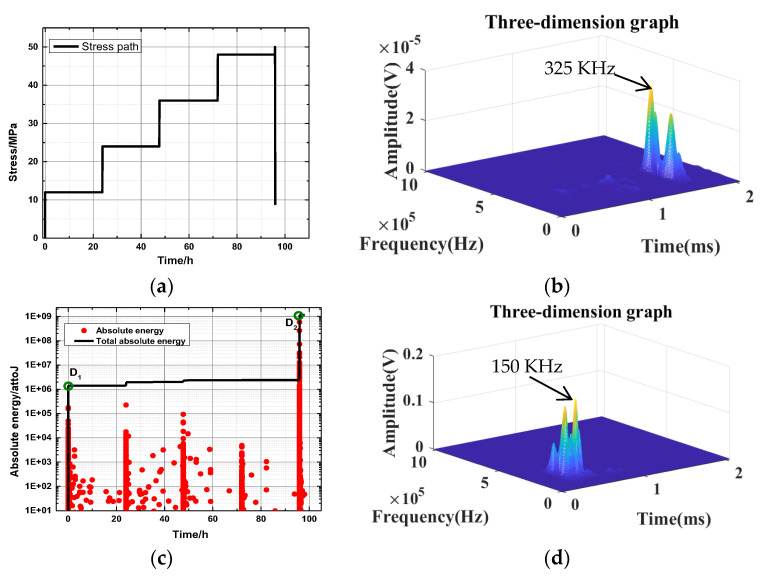
Stress with time for a 45-degree bedding angle under the saturated state: (**a**) the actual stress path; and (**c**) the corresponding acoustic emission energy curve. Images (**b**,**d**) are the waveform characteristics of key points.

Stress with time for a 45-degree bedding angle under the dry state is taken as a typical example, as shown in Figure 12. Table 7 shows that the accumulated energy level of D_1_ and the key point of different bedding dip angles in the dry state is between 6.4 × 10^5^ and 5.5 × 10^6^. The cumulative energy level of key point D_2_ is between 3.3 × 10^8^ and 3.4 × 10^10^. Table 8 shows that the key points D_1_ to D_2_ of rock samples with different bedding angles in the dry state change from low amplitude to high amplitude, and the amplitude increases significantly.

### 4.3. Summary and Discussion

**Figure 13:**
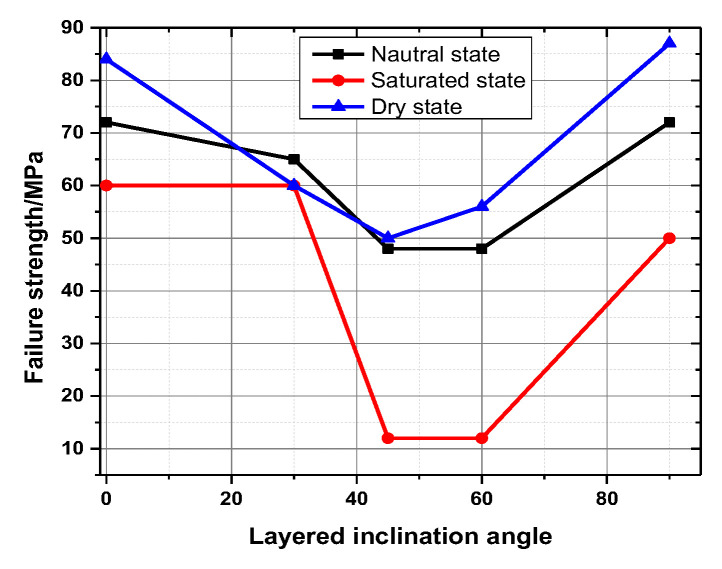
Creep failure strength with layered angles.

According to the analysis, creep failure strength and frequency value are the key parameters for creep characteristics of layered rock masses. Therefore, creep failure strength and frequency value are the values selected for analysis. Figure 13 shows the creep failure strength with the layered angle. Under the same water-bearing conditions, the long-term strength of the rock sample decreases with increasing bedding dip. Under the conditions of the same bedding dip, the long-term strength of the rock at failure decreases with increasing water content.

**Figure 14:**
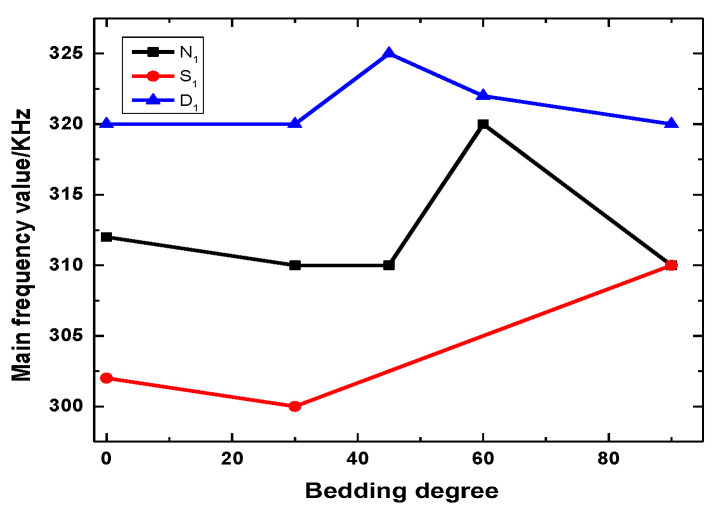
Curves of initial main frequency value with different bedding angles under different water content levels.

Figure 14 shows the curves of the initial main frequency value with different bedding angles under varying water contents. The principal frequency value at the initial moment is not significantly affected by the structural effect, but the influence of water on the principal frequency value at the initial moment of rock samples is very significant because the higher the water content is, the more large-scale cracks are generated.

**Figure 15:**
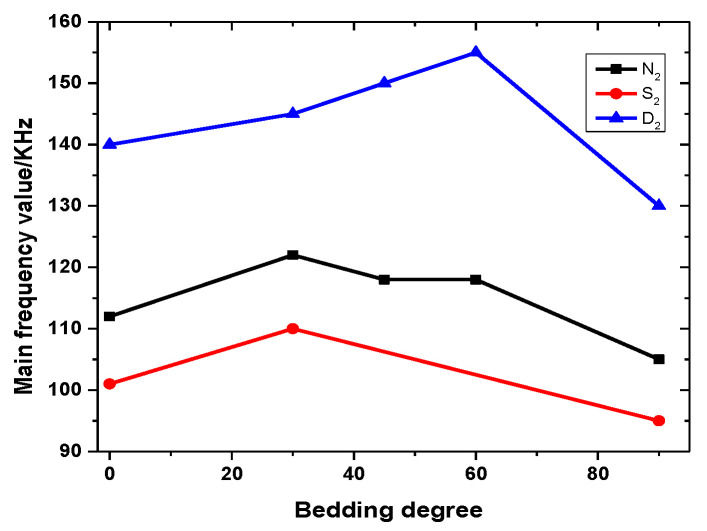
Curves of final main frequency value with different bedding angles under different water content levels.

Figure 15 shows the curves of the final main frequency value with different bedding angles under various water contents. The influence of the structural effect on the principal frequency value at failure time is mainly reflected in the trend that the principal frequency value increases first and then decreases with the increment in bedding angle. However, most rock samples with bedding dip angles of 30°, 45°, and 60° mainly exhibit shear failure, and large-scale cracks are relatively few. The influence of water content on the main frequency value of rock samples is very significant. With the increase in water content, the main frequency value of the rock samples with the same bedding angle has a tendency to decrease gradually.

### 4.4. Strain–Time Result Analysis

#### 4.4.1. Analysis of Rock Sample Creep Time

**Figure 16:**
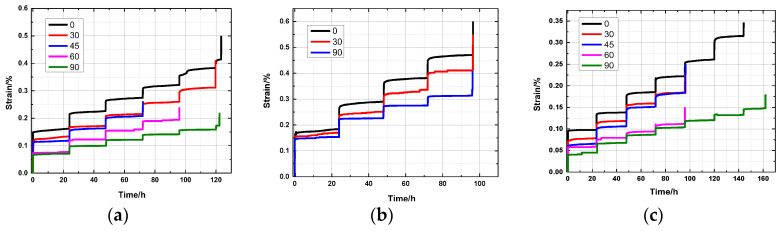
Strain with time for different bedding angles under different states: (**a**) natural state; (**b**) saturated state; and (**c**) dry state.

The total creep time of rock samples with different bedding dip angles in the same water-bearing state is analyzed under the action of various stress levels (Figure 16). The rock samples with bedding dip angles of 45° and 60° have short durations. Taking the natural state bedding dip angle of 45° as an example, the shared time from the initial loading to the final destruction was 72.0 h. The rock samples with bedding dip angles of 0° and 90° lasted a long time. Among them, the rock samples with a bedding dip angle of 90° in the dry state lasted the longest, and the time from the initial loading to the final failure was 161.8 h. The analysis of the total creep time of the rock samples with the same bedding dip in different water-bearing states under different stress levels shows that with the increase in water content, the creep time of the rock sample decreases, and the rock sample with a bedding dip of 45° in the saturated state takes the shortest time or approximately 0.1 h.

#### 4.4.2. Analysis of Instantaneous Creep of Rock Samples

Rock samples with the same water-bearing state and different bedding angles produce large instantaneous strains during initial loading compared with all levels. The first stress level of the rock sample loads has the largest instantaneous strain, which is the result of the original pores, cracks, and mineral particles being compacted in the sample. As the stress level increases, the instantaneous creep value tends to decrease. Under the action of the same bedding angle and stress level, the instantaneous creep of the rock sample increases with the water content. The softening of water causes the large microcracks in the rock sample, which increases the creep.

#### 4.4.3. Comparison of the Instantaneous Elastic Modulus of the Rock Samples

The ratio of each level of stress increment to the instantaneous strain increment under the action of this stress level is used as the instantaneous elastic modulus of this stress level. The instantaneous elastic moduli of rock samples with different water-bearing states and different bedding angles are shown in Table 9,Table 10,Table 11. The instantaneous elastic modulus of the rock samples increases with the stress level. It increases faster when the stress level is low and increases slowly when the stress level is high. The comparison of the rock samples with the same water-bearing state and different bedding dip angles suggests that the instantaneous elastic modulus tends to increase with the dip angle under the same stress level. The reason is that the loading direction is gradually parallel to the bedding direction as the bedding angle increases. The compaction angle of the rock sample gradually decreases, which is manifested as an instantaneous decrease in strain and increase in elastic modulus.

#### 4.4.4. Comparison of Creep Strain of Rock Samples

**Figure 17:**
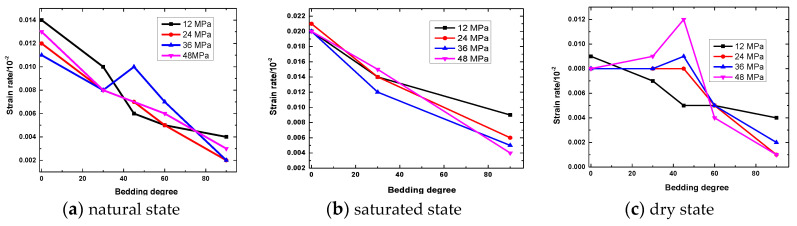
Creep strain with bedding angle of rock sample under different states.

The creep process of a rock sample can be divided into instantaneous strain and creep strain. In view of the different stress levels when the rock samples with different bedding dip angles fail, the creep strain that corresponds to the first four levels of stress is selected for research, which is the same as that in Figure 17. The trend of the creep strain with bedding dip angle under the action of various stress levels in the water-bearing state is presented. The overall creep strain of the rock sample is relatively small. The creep strains of the slate in the natural, saturated, and dry states are between 0.002% and 0.014%, 0.004% and 0.021%, and 0.001% and 0.012%, respectively. Under the same water-bearing state and stress level, the amount of creep strain of a rock sample gradually decreases with the increase in bedding dip.

#### 4.4.5. Analysis of the Characteristics of Creep Deformation of Rock Samples

Overall, the creep deformation of the rock sample under middle and low stress levels is mainly divided into transient creep and steady-state creep. The creep rate decreases rapidly within 1.5 h of loading. The creep rate of the rock sample in the steady-state creep stage remains stable, the creep rate is low, and the amount of creep is small. At a higher stress level, the long-term strength of the rock sample is reached, the deformation increases rapidly, and failure occurs in a very short period of time. The creep failure process does not significantly accelerate the creep process, thereby showing the characteristics of brittle failure and a macroscopic fracture surface.

#### 4.4.6. Constitutive Model

According to the experimental results, the Burgers model can reflect the creep characteristics of the slate well. The creep equation of the Burgers model is expressed as follows:

(5) $$\varepsilon =\frac{\sigma }{G_{1}}+\frac{\sigma }{\eta_{1}}t+\frac{\sigma }{G_{2}}(1-e^{-\frac{G_{2}}{\eta_{2}}t}),$$

where *G_1_* and *G_2_* are the elastic modulus of the elastic element, and *η_1_* and *η_2_* are the viscosity coefficients of the viscous element.

The determination of creep model parameters is complicated because of the large number of parameters. The leastsquares method is used to perform parameter inversion analysis with MATLAB by curving fitting tools to determine the model’s parameters. In view of the large number of rock samples and similar analysis methods, rock samples with a natural bedding angle of 0° were selected as typical rock samples for in-depth analysis, and the parameter inversion results are shown in Table 12.

**Figure 18:**
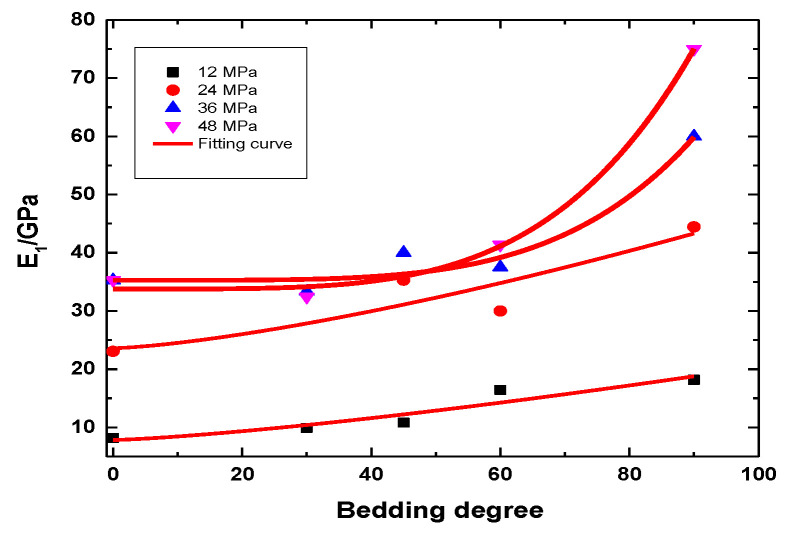
Elastic modulus with bedding angle for slate under the natural state.

Figure 18 shows that the instantaneous elastic modulus of rock samples under the natural state increases nonlinearly with the bedding angle, and the relationship among the instantaneous elastic modulus of all loading levels and bedding angle is expressed as:

(6) $$G(\partial )=a\partial^{b}+c,\;$$

where *a*, *b*, and *c* are the fitting parameters, and ∂ is the bedding angle.

**Figure 19:**
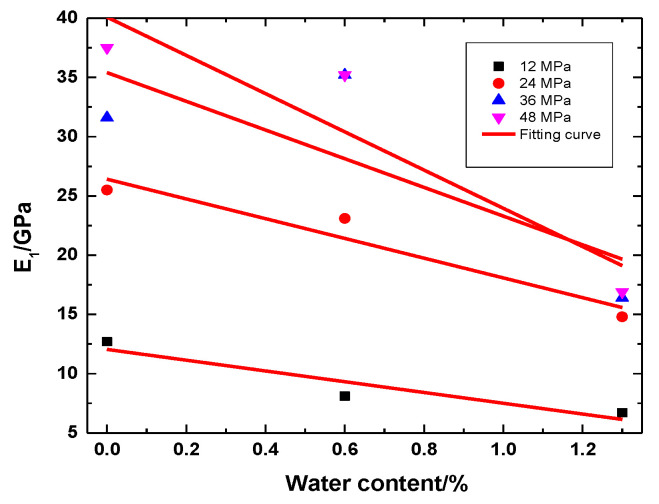
Elastic modulus with water content of 0° for slate.

The fitting curves of the instantaneous elastic modulus and water content of rock samples with a bedding angle of 0° in different water bearing states are shown in Figure 19. The instantaneous elastic modulus decreases linearly with the increase in water content under different stress levels. The instantaneous elastic modulus and water content are expressed as follows:

(7) G(w)=dw+e

where *d* and *e* are the fitting parameters, and *w* is the moisture content.

Ignoring the influence of the coupling between the bedding dip angle and the water content on the instantaneous elastic modulus, and considering the influence of the bedding angle and water content on the elastic modulus of rock samples based on Formulas (6) and (7), the relationship between elastic modulus and bedding angle and water content can be written as follows:

(8) $$G_{1}=\rho_{1}G(\partial )+\rho_{2}G(w)=a\rho_{1}\partial^{b}+d\rho_{2}w+c\rho_{1}+e\rho_{2},$$

where *ρ_1_* and *ρ_2_* are the influence rates of the bedding dip angle and water content on the instantaneous elastic modulus, respectively.

By substituting (8) into (5), the creep equation of the slate that reflects the water content and structural effect can be written as follows:

(9) $$\varepsilon =\frac{\sigma }{a\rho_{1}\partial^{b}+d\rho_{2}w+c\rho_{1}+e\rho_{2}}+\frac{\sigma }{\eta_{1}}t+\frac{\sigma }{G_{2}}(1-e^{-\frac{G_{2}}{\eta_{2}}t}),$$

where *a*, *b*, and *c* are the fitting parameters and the bedding dip angles. *d* and *e* are the fitting parameters, and w is the moisture content. *ρ_1_* and *ρ_2_* are the influence rates of the bedding dip and water content on the instantaneous elastic modulus, respectively.

## 5. Conclusions

The change law of variable mechanical properties of slate is revealed, a creep constitutive model that considers the water content and structural effects is established, and the water absorption and softening mechanism of slate are studied through microstructure scanning and creep experiments considering the two influencing factors of water content and structural effect. The main conclusions are drawn as follows:

(1) With the decrease in water content, the long-term strength of the same bedding rock sample increases, and the damage worsens. Under the same water-bearing conditions, the rock samples with bedding dip angles of 0°, 30°, and 90° have great long-term strength and severe failure, whereas rock samples with bedding dip angles of 45° and 60° have low long-term strength and mild failure.
(2) Under the same water content, the initial energy release increases with the bedding angle. Under the same water content, the energy release during failure first decreases, and then increases with the bedding angle. With the increase in water content, the initial energy, the cumulative energy at the time of failure, the initial main frequency, and the main frequency at the time of failure tend to decrease.
(3) The creep deformation of rock samples under the action of medium and low stress levels can be divided into transient and steady-state creep, respectively. In the steady-state creep stage, the creep rate of the rock sample remains basically stable, and the creep rate is low, and the amount is small. At higher stress levels, the long-term strength of the rock sample is reached, the deformation increases rapidly, and failure occurs in a very short period of time. The creep failure process does not significantly accelerate the creep process, thereby showing the characteristics of brittle failure and a macroscopic fracture surface. According to the results, danger zones are designated as those with high water content and a 45° bedding angle.

## Figures and Tables

**Table 1:** Creep experiment scheme.

Scheme	State	Bedding Angle (°)				
A	Natural	0	30	45	60	90
B	Saturated	0	30	45	60	90
C	Dry	0	30	45	60	90

**Table 2:** Level of stress.

	First Level	Second Level	Second Level	Third Level	Fourth Level	Fifth Level	Sixth Level
Duration/h	24	24	24	24	24	24	24
Stress/MPa	12	24	36	48	60	72	84

**Table 3:** AE energy levels for different bedding angles under natural state.

Key Point	0°	30°	45°	60°	90°
	AE Energy/aJ	AE Erengy/aJ	AE Erengy/aJ	AE Erengy/aJ	AE Erengy/aJ
N_1_	4.4 × 10^4^	3.9 × 10^4^	1.3 × 10^6^	6.6 × 10^5^	3.3 × 10^5^
N_2_	8.2 × 10^8^	2.5 × 10^8^	4.0 × 10^8^	2.4 × 10^8^	1.8 × 10^9^

**Table 4:** Dominant frequency value of key point for different bedding angles under natural state.

Key Point	0°	30°	45°	60°	90°
	DFV/KHz	DFV/KHz	DFV/KHz	DFV/KHz	DFV/KHz
N_1_	312	310	310	320	310
N_2_	112	122	118	118	105

**Table 5:** AE energy level for different bedding angle under saturated state.

Key Point	0°	30°	90°
	AE Erengy/aJ	AE Erengy/aJ	AE Erengy/aJ
S_1_	2.7 × 10^3^	8.6 × 10^3^	3.6 × 10^4^
S_2_	6.4 × 10^7^	4.1 × 10^7^	1.1 × 10^8^

**Table 6:** Dominant frequency value of key point for different bedding angles under saturated state.

Key Point	0°	30°	90°
	DFV/KHz	DFV/KHz	DFV/KHz
S_1_	302	300	310
S_2_	101	110	95

**Table 7:** AE energy level for different bedding angles under dry state.

Key Point	0°	30°	45°	60°	90°
	AE Erengy/aJ	AE Erengy/aJ	AE Erengy/aJ	AE Erengy/aJ	AE Erengy/aJ
D_1_	6.4 × 10^5^	1.3 × 10^6^	1.4 × 10^6^	5.5 × 10^6^	3.9 × 10^6^
D_2_	1.7 × 10^9^	3.3 × 10^8^	1.1 × 10^8^	1.3 × 10^9^	3.4 × 10^10^

**Table 8:** Dominant frequency values of key point for different bedding angles under dry state.

Key Point	0°	30°	45°	60°	90°
	DFV/KHz	DFV/KHz	DFV/KHz	DFV/KHz	DFV/KHz
D_1_	320	320	325	322	320
D_2_	140	145	150	155	130

**Table 9:** Instantaneous elastic modulus of rock samples in a natural state at different stress levels.

Bedding Angles	12 MPa	24 MPa	36 MPa	48 MPa	60 MPa	72 MPa
0	8.1	23.1	35.2	35.2	35.2	39.1
30	9.9	36.4	33.3	32.4	32.5	-
45	10.8	35.3	40	-	-	-
60	16.5	30.0	37.5	41.4	-	-
90	18.2	44.4	60	75	76.9	77.8

**Table 10:** Instantaneous elastic modulus of rock samples in a saturated state at different stress levels.

Bedding Angles	12 MPa	24 MPa	36 MPa	48 MPa
0	6.7	14.8	16.4	16.9
30	8.3	19.0	20.0	21.1
90	8.4	17.6	26.7	35.3

**Table 11:** Instantaneous elastic modulus of rock samples in a dry state at different stress levels.

Bedding Angles	12 MPa	24 MPa	36 MPa	48 MPa	60 MPa	72 MPa
0	12.7	25.5	31.6	37.5	40.0	26.1
30	16.9	37.5	33.3	54.5	-	-
45	20.0	35.3	31.6	37.5	-	-
60	21.1	75.0	120.0	92.3	-	-
90	30.5	109.1	70.6	80.0	92.3	100.0

**Table 12:** Parameter inversion results of 0° bedding angle for slate under the natural state.

Stress Level/MPa	*G*_1_	*G*_2_	*η* *_1_*	*η* *_2_*	Coefficient of Correlation *R*^2^
12	56.8	9.4	1668.6	0.2	0.94
24	14.9	41.8	7018.4	1.5	0.98
36	17.4	61.5	8343.9	3.4	0.99
48	17.5	127.9	11748.5	5.0	0.98
60	19.1	121.8	6317.5	17.3	0.96

## Data Availability

All data and models generated or used during the study appear in the submitted article.
